# Novel Gyroviruses, including Chicken Anaemia Virus, in Clinical and Chicken Samples from South Africa

**DOI:** 10.1155/2014/321284

**Published:** 2014-04-29

**Authors:** Heidi E. M. Smuts

**Affiliations:** Division Medical Virology/National Health Laboratory Service, Department of Clinical Sciences, Faculty of Health Sciences, University of Cape Town, Observatory 7925, South Africa

## Abstract

*Introduction*. Chicken anaemia virus, CAV, was until recently the only member of the *Gyrovirus* genus. 6 novel gyroviruses, AGV2, HGyV1, and GyV3-6, have since been discovered in human and chicken samples. *Methods*. PCR amplification of the VP2 gene was used to detect AGV2/HGyV1, GyV3, and CAV in a range of clinical samples including stool, respiratory, CSF, and HIV-positive plasma. Screening of fresh local chicken meat was also performed. *Results*. AGV2/HGyV1 or GyV3 was detected in stools from healthy children (17/49, 34.7%) and patients with diarrhoea (22/149, 14.8%). 1.2% (3/246) nasopharyngeal respiratory samples were positive. No AGV2/HGyV1 or GyV3 was detected in nasal swabs from wheezing patients, in CSF from patients with meningitis, and in HIVpositive plasma. CAV was found in 51% (25/49) of stools from healthy children and 16% (24/149) in diarrhoea samples. Screening of 28 chicken samples showed a higher prevalence of gyrovirus (20/28, 71%) compared to CAV (1/28, 3.6%). Phylogenetic analysis of the CAV VP1 gene showed South African sequences clustering with Brazilian isolates from genotypes D2 and A2. *Conclusion*. Novel gyroviruses, including CAV, are present in the South African population with diarrhoea and respiratory illness as well as in healthy children. Their presence suggests an origin from chicken meat consumption.

## 1. Introduction

Until recently chicken anaemia virus (CAV) was the only member of the genus* Gyrovirus* in the Circoviridae family. This genus is characterized by small nonenveloped DNA viruses with a negative sense single-stranded circular DNA of about 2.3 kb [1]. Circoviruses, in contrast, have an ambisense genome. The similarity of the gyrovirus genome organization to annelloviruses, with 3 overlapping open reading frames (ORFs), has led to the recommendation that gyroviruses become a subfamily, Gyrovirinae, within the Anelloviridaefamily [2].

In early 2011 Rijsewijk et al. [3] reported the discovery of a distant relative to CAV, avian gyrovirus 2 (AGV2), in diseased chicken from Brazil, with only 40% homology to CAV. Later that year, Sauvage et al. [4] identified a very closely related gyrovirus on human skin (HGyV1). Subsequently 4 other novel gyroviruses have been described. Gyrovirus 3 (GyV3) was identified by viral metagenomics in faeces from Chilean children with acute gastroenteritis and also in chicken meat [5]. A phylogenetically distinct gyrovirus (GyV4) was also discovered in both human stool samples and chicken meat by 454 pyrosequencing [6]. Further 2 divergent gyroviruses, GyV5 and GyV6, were found in the stools of Tunisian children with diarrhoea [7].

CAV is an economically important pathogen in the poultry industry causing severe anaemia and immunosuppression in young 2-3-week-old chicken that lack protective maternal antibodies [8]. In adult chickens CAV infection is subclinical, but financial losses may be incurred by poultry farmers due to lack of weight gain and susceptibility to secondary infections.

The role of CAV in the African poultry context has been addressed by a number of researchers [9–16]. Seroprevalence in Nigeria, Egypt, Central African Republic, and Cameroon ranged from 37% to 89% depending on age of chicken. Further, CAV DNA could be detected in the majority (77%) of seropositive chickens, indicating persistent virus shedding in the presence of antibodies. To date there has only been one brief report on CAV in South African chickens [17], where 3 broiler chickens were seropositive.

The aim of this study was to determine if some of the novel gyroviruses and CAV are present in the South African population and in chickens from this region.

## 2. Materials and Methods

### 2.1. Clinical Samples

Anonymized stool samples from 49 healthy children, aged 6–36 months, were obtained with parental/guardian consent from a crèche over 2 periods: summer (February/March 2006) and winter (July/August 2006). Stool samples from patients with diarrhoea submitted to the virology and microbiology diagnostic laboratory over the same time periods in 2006 were also examined (*n* = 149).

246 nasopharyngeal respiratory samples from children aged 1–60 months previously screened for the 7 common respiratory viruses (adenovirus, human respiratory syncytial virus, human metapneumovirus, influenza A and B, parainfluenza virus 1–3, and human rhinovirus A) were tested, of which 152 were negative and the remainder (*n* = 94) were positive for one of the above viruses.

Nasal swabs (*n* = 48) collected in May 2004 from children aged 6–25 months with acute wheezing were also screened. Informed consent from a parent or guardian had been obtained.

The presence of gyrovirus in plasma from HIV-infected patients (*n* = 48) was evaluated as Maggi et al. [18] had identified HGyV1 in one Italian patient. The CD4/*μ*L count ranged from 13 to 1065 with a mean of 397.

94 cerebrospinal fluid (CSF) samples from patients with suspected meningitis were also screened.

### 2.2. Chicken Meat

Fresh chicken meat for human consumption was purchased in August 2012 from 4 retail stores in Cape Town, South Africa. These included 4 thighs and 4 drum sticks from 3 of 4 stores and 4 thighs from the remaining store.

A sterile scalpel was used to remove a small piece of the skin and underlying meat from each sample. This was placed in a sterile 1.5 mL Eppendorf tube and stored at −20°C until extracted.

### 2.3. DNA Extraction

Total nucleic acid was extracted from nasopharyngeal respiratory samples using the MagNA Pure LC automated extraction method as per manufacturer's instructions (Roche Diagnostics GmbH, Penzberg, Germany). Nucleic acid was extracted from stool, nasal swab, CSF, plasma, and chicken meat samples using the QIAamp DNA stool mini kit and QIAamp DNA mini kit, with protocols appropriate for body fluid or tissue as per manufacturer's instructions (Qiagen, Hilden, Germany).

### 2.4. PCR for Detection of Gyrovirus or CAV

Consensus primers targeting the conserved VP2 region of the gyroviruses, CAV, AGV2/HGyV1, GyV3, and GyV6, were used as outer primers as described by Phan et al. [5]. Nested primers specific for either CAV (CAV F1n 5′ GGCAGTGAATCGGCGCTTAGCCG and CAV R1n 5′ AGTCGCTTGAGGTGGTGCCACCG) or AGV2/HGyV1 and GyV3 (ConGy F1n 5′ GGCAGTGAATTGCCGCTTAGGC and ConGy R1n 5′ CGCAGTCTGTGTCTCCAGTGC) were used to improve sensitivity. The inner PCR product was ~550 bp in size. The PCR conditions for the first round were as follows: denaturation at 95°C for 3 minutes, 40 cycles of 95°C for 15 sec, 45°C for 25 sec, and 72°C for 35 sec followed by a final 72°C elongation step for 7 min. The second round of amplification was the same as above with an increase in the annealing temperature to 55°C. PCR products were visualised by electrophoresis through a 2% agarose gel, ethidium bromide staining, and UV illumination. GyV4, GyV5, and GyV6 are not amplified with the nested primers and therefore this study did not measure their prevalence.

### 2.5. Sanger Sequencing

The VP2 amplicons were sequenced directly in both directions using either CAV or GyV nested PCR primers. The BigDye terminator cycle sequencing kit was used (Applied Biosystems, Foster City CA, USA). The VP2 genes were aligned with reference sequences from GenBank using ClustalW [19] and phylogenetic trees constructed using MEGA 5.05 [20] with 1000 bootstrap resamplings. BLAST analysis was also undertaken.

Improved phylogenetic resolution of CAV positive samples was investigated by amplification of the 1349 bp of the VP1 and overlapping VP2 and VP3 genes using primers previously described by Natesan et al. [21] and Eltahir et al. [22]. Increased sensitivity was achieved by the design of a nested VP1 forward primer (5′CCGCAAGAAGTATAAGAC).

## 3. Results

### 3.1. Gyrovirus in Clinical Samples

AGV2/HGyV1 or GyV3 was detected in stools from both healthy children (17/49, 34.7%) and patients with diarrhoea (22/149, 14.8%) (Table 1). The detection rate in respiratory samples was considerably lower at 1.3% (2/152) in samples negative for the common respiratory viruses and 1.1% (1/94) in positive samples (Table 1). In the latter sample, parainfluenza 1–3 was previously detected using the Seeplex RV7 assay (Seegene, Seoul, South Korea). No AGV2/HGyV1 or GyV3 was detected in the nasal swabs from children with acute wheezing, in plasma from HIV-infected individuals, or in the CSF from patients with suspected meningitis.

### 3.2. CAV in Stool Samples

CAV was detected in 51% (25/49) stool samples from healthy children and 16.1% (24/149) in diarrhoea samples (Table 1). Nasopharyngeal respiratory samples, nasal swabs, HIV-positive plasma, and CSF were not screened for the presence of CAV.

Dual infection with both CAV and AGV2/HGyV1/GyV3 was found in 10/49 (20.4%) and 8/149 (5.4%) stools from healthy children and patients with diarrhoea, respectively. Codetection of CAV and AGV2/HGyV1/GyV3 was not statistically linked (*P* = 0.85).

### 3.3. CAV/Gyrovirus in Chicken Meat

Screening of 28 chicken samples for the presence of CAV DNA or AGV2/HGyV1/GyV3 DNA showed a considerably higher prevalence of gyrovirus (20/28, 71.4%) compared to CAV (1/28, 3.6%). From one store, C, 7/8 samples were negative for both gyrovirus and CAV, while chicken meat obtained from the remaining 3 stores was positive for AGV2/HGyV1, with only one sample coinfected with CAV (Table 1).

### 3.4. Phylogenetic and Amino Acid Analysis

**Figure 1:**
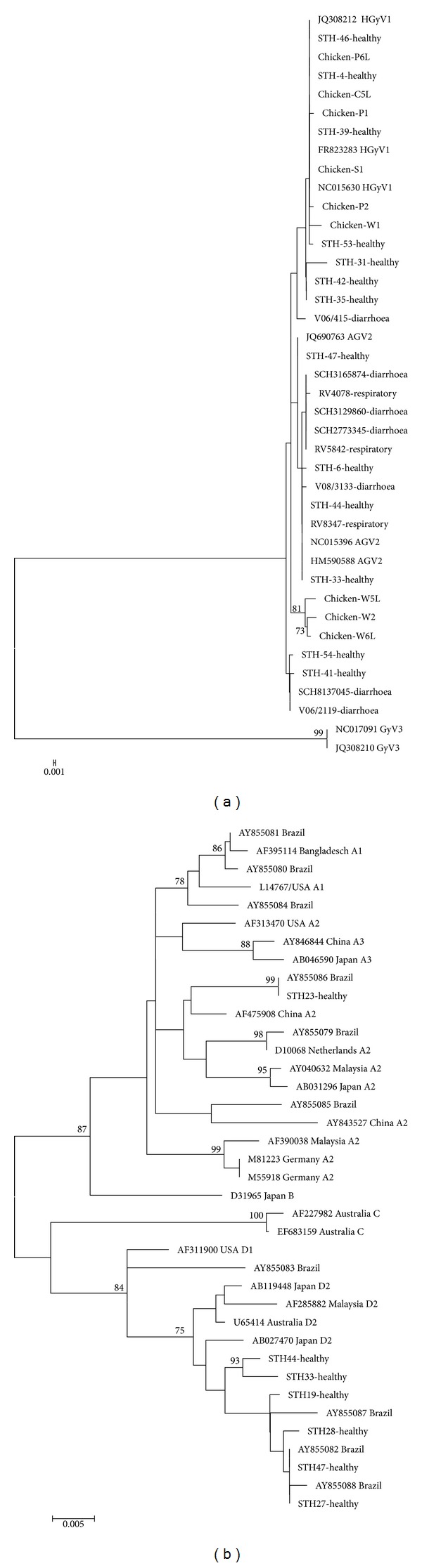
Phylogenetic trees generated by neighbour-joining analysis of the VP2 region of AGV2, HGyV1, and GyV3 (a) and CAV VP1 (b) of South African isolates of human and chicken origin. Reference sequences of AGV2, HGyV1, GyV3, and CAV were retrieved from the NCBI GenBank database and are indicated by accession numbers and country of origin. Bootstrap values greater than 75% are indicated at the nodes of the tree. The branch lengths are proportional to the evolutionary distances as shown on the scale.

Phylogenetic analysis of the VP2 region of samples successfully sequenced showed the presence of only AGV2/HGyV1, with 56.3% (18/32) grouping with AGV2 sequences and 43.5% (14/32) with HGyV1 sequences (Figure 1(a)). No GyV3 was detected. All 3 respiratory samples clustered with AGV2 sequences. Of the chicken samples successfully sequenced, 6/9 had sequence homology to HGyV1 and the remaining 3 samples homology with AGV2 sequences.

The VP1 region of CAV provided phylogenetic information for the classification of isolates into different genotypes (Figure 1(b)). Analysis of the 1349 bp region showed that 6/7 CAV-positive samples grouped together with genotype D, particularly D2 isolates from USA, Japan, Malaysia, and Australia. The remaining sample clustered within genotype A, but formed a separate lineage with a high bootstrap value (data not shown). Phylogenetic analysis of a truncated VP1 region (481 bp) to allow inclusion of VP1 sequences from Brazil (AY855079-88) retained the phylogeny as determined using the larger VP1 region, but now showed that all South Africa isolates clustered with the Brazilian sequences of either genotype A or D (Figure 1(b)).

Analysis of the deduced 410 amino acids of CAV VP1 protein revealed that no amino acids were characteristic of the South African isolates, although a proline (P) at position 286 was found in all sequences and in common with 2 isolates from Japan and 1 each from Australia and Malaysia. The region responsible for virulence [23] showed a glutamine (Q) at position 394 instead of histidine, indicative of a highly virulent strain (data not shown).

## 4. Discussion

The recent identification of novel gyroviruses in the human population led to the screening of a range of clinical samples from patients and healthy children from South Africa. The present study confirms the presence of AGV2/HGyV1 and CAV in this region. GyV3 was not detected and this study did not test for GyV4, GyV5, and GyV6. As limited studies on gyroviruses in healthy and sick individuals are available, the pathogenesis and clinical importance are not well understood. HGyV1 may be a member of the skin virome [4], although Chu et al. [6] were unable to detect gyroviruses in skin swabs from healthy individuals. Novel gyroviruses have been detected in stools from patients with diarrhoea with a prevalence of 1–9.3%, while no gyrovirus was detected in stools from healthy individuals [5, 6]. The present study showed a higher prevalence of gyroviruses (AGV2/HGyV1) in stools from patients with diarrhoea (15%), while 35% of healthy children also had evidence of AGV2/HGyV1 DNA. Chu et al. [6] demonstrated that gyroviruses were more commonly detected in patients younger than 20 years of age.

Viruses found in the stool samples may also play a role in respiratory and systemic diseases. This was investigated by screening respiratory samples from young patients hospitalized with respiratory illness and children attending an outpatient hospital clinic with acute wheezing. AGV2 was detected in 3 hospitalized patients, with one patient coinfected with parainfluenza virus 1–3. Further studies are required to determine if gyroviruses can cause respiratory infections requiring hospitalization.

Gyrovirus was not detected in blood from HIV-infected individuals, although other studies have reported a low prevalence in both blood healthy donors [24] and immunocompromised HIV-positive and kidney transplant patients [18]. Our results may reflect the small number of samples screened.

Circoviruses are able to cross the blood-brain barrier and cause infections of the central nervous system. Porcine circovirus type 2 infection in piglets results in cerebellar vasculitis [25], while in humans TTV and novel cycloviruses were found in the CSF of patients with subacute dementia, unexplained paraplegia, and acute CNS infections of unknown aetiology [26–28]. Screening of CSF samples from patients with possible meningitis showed no evidence of gyrovirus infection.

CAV was detected more frequently in stools from healthy children (>50%) compared to those with diarrhoea (16%). Prior studies by Phan et al. [5] and Chu et al. [6] also reported a high CAV prevalence in faecal samples from healthy donors of 25% and 53%, respectively. This high detection rate suggests that consumption of infected chicken meat may possibly play a role. However, in this report screening of fresh chicken meat purchased from a number of different stores in the study area does not support this assumption, as only one positive CAV sample was found. In contrast, a significant proportion of chicken meat was positive for AGV2/HGyV1. This discrepancy may be explained by the fact that only fresh chicken meat purchased in 2012 was screened, while the clinical samples were collected from 2004–2006 when imported frozen chicken products were more readily available for consumption.

A number of live attenuated CAV vaccines are available and used in the poultry industry. However, evidence of this practice occurring in the South African poultry industry is not readily available. If undertaken, breeder hens are vaccinated to obtain high levels of circulating antibodies which can be passed onto progeny preventing early infections and disease caused by CAV (personal communication, Dr. Nkuna, South African Poultry Association).

From 2001 a significant amount of in-bone frozen chickens were imported from Brazil, which in 2010 accounted for over 70% of the South African import market of chickens [29]. This dropped substantially since 2012 with the introduction of stricter antidumping legislation. These imported products were significantly cheaper than the locally sourced chickens and may have been preferentially purchased and consumed by a large proportion of the South African population. Simionatto et al. [30] reported that CAV was detected in 90% of field samples collected from commercial breeders, broilers, and free-range chickens from different regions of Brazil. Whether these chickens are infected with CAV either naturally or through vaccination programs is not known.

Phylogenetic analysis of the VP1 region provides further evidence that CAV detected in this study may originate from Brazil. All the South African isolates, of either genotype D or A, clustered together with Brazilian isolates. Sourcing of imported chicken products from the earlier time of human sample collection to confirm this association was not possible in 2013.

## 5. Conclusion

The role and clinical relevance of gyroviruses in human disease require further investigation. Larger studies on patients with a variety of medical conditions are needed to elucidate the pathogenesis of gyrovirus infection.

## Figures and Tables

**Table 1:** PCR screening of clinical and chicken samples for the presence of AGV2/HGyV1 and CAV.

Samples	AGV2/HGyV1 Number (%)	CAV Number (%)
Control stool	17/49 (34.7%)	25/49 (51%)
Diarrhoea stool	22/149 (14.8%)	24/149 (16.1%)
Negative respiratory virus NPA	2/152 (1.3%)	N/D
Positive respiratory virus NPA	1/94 (1.1%)	N/D
HIV-positive plasma	0/48	N/D
Acute wheezing nasal swabs	0/48	N/D
Meningitis CSF	0/94	N/D
Chicken meat store P	7/8 (87.5%)	0/8
Chicken meat store W	8/8 (100%)	0/8
Chicken meat store C	1/8 (12.5%)	0/8
Chicken meat store S	4/4 (100%)	1/4 (25%)

NPA: nasopharyngeal aspirates; N/D: not done.
